# Costs of introducing pneumococcal, rotavirus and a second dose of measles vaccine into the Zambian immunisation programme: Are expansions sustainable?

**DOI:** 10.1016/j.vaccine.2016.06.050

**Published:** 2016-07-29

**Authors:** Ulla Kou Griffiths, Fiammetta Maria Bozzani, Collins Chansa, Anthony Kinghorn, Penelope Kalesha-Masumbu, Cheryl Rudd, Roma Chilengi, Logan Brenzel, Carl Schutte

**Affiliations:** a Department of Global Health and Development, London School of Hygiene & Tropical Medicine, 15-17 Tavistock Place, London WC1H 9SH, United Kingdom; b Strategic Development Consulting (SDC), Pietermaritzburg, South Africa; c Perinatal Health Research Unit, University of the Witwatersrand, Chris Hani Baragwanath Hospital, Soweto, South Africa; d World Bank, Zambia Country Office, Banc ABC House, Church Road, Lusaka, Zambia; e World Health Organization, Lusaka, Zambia; f Center for Infectious Disease Research in Zambia (CIDRZ), Plot 5032 Great North Road, Lusaka, Zambia; g Bill and Melinda Gates Foundation, 1300 I Street, NW Suite 200, Washington, DC, United States

**Keywords:** Gavi, Vaccines, Financing, Affordability, Costs

## Abstract

**Background:**

Introduction of new vaccines in low- and lower middle-income countries has accelerated since Gavi, the Vaccine Alliance was established in 2000. This study sought to (i) estimate the costs of introducing pneumococcal conjugate vaccine, rotavirus vaccine and a second dose of measles vaccine in Zambia; and (ii) assess affordability of the new vaccines in relation to Gavi’s co-financing and eligibility policies.

**Methods:**

Data on ‘one-time’ costs of cold storage expansions, training and social mobilisation were collected from the government and development partners. A detailed economic cost study of routine immunisation based on a representative sample of 51 health facilities provided information on labour and vaccine transport costs. Gavi co-financing payments and immunisation programme costs were projected until 2022 when Zambia is expected to transition from Gavi support. The ability of Zambia to self-finance both new and traditional vaccines was assessed by comparing these with projected government health expenditures.

**Results:**

‘One-time’ costs of introducing the three vaccines amounted to US$ 0.28 per capita. The new vaccines increased annual immunisation programme costs by 38%, resulting in economic cost per fully immunised child of US$ 102. Co-financing payments on average increased by 10% during 2008–2017, but must increase 49% annually between 2017 and 2022. In 2014, the government spent approximately 6% of its health expenditures on immunisation. Assuming no real budget increases, immunisation would account for around 10% in 2022. Vaccines represented 1% of government, non-personnel expenditures for health in 2014, and would be 6% in 2022, assuming no real budget increases.

**Conclusion:**

While the introduction of new vaccines is justified by expected positive health impacts, long-term affordability will be challenging in light of the current economic climate in Zambia. The government needs to both allocate more resources to the health sector and seek efficiency gains within service provision.

## 1 Introduction

Gavi, the Vaccine Alliance was founded in 2000 and is now the largest external funding source for vaccines in low- and lower middle-income countries [1]. Introducing new vaccines requires substantial investments, not only in vaccine supplies, but also in ‘systems costs’, such as cold chain expansions [2], [3]. Cost estimates of new vaccine introduction are vital both to Gavi and to recipient countries [4].

Zambia has introduced four new vaccines with Gavi support. The combined diphteria-tetanus-pertussis (DTP)-*Haemophilus influenzae* type B (Hib) vaccine was introduced in 2004. This was switched to DTP-hepatitis B-Hib (‘pentavalent’) vaccine in 2005. In 2009, a proposal was submitted for pneumococcal conjugate vaccine (PCV), rotavirus vaccine (RV) and a second dose of measles (MSD). Gavi approved PCV and MSD in 2010 and RV in 2011, following evidence of plans for cold chain expansions. However, PCV and MSD were only introduced in July 2013 and RV in November 2013. Delays were due to a measles outbreak in 2012, relocation of the Child Health Unit from the Ministry of Health to the new Ministry of Community Development, Mother and Child Health in 2011, delays in receiving the Gavi vaccine introduction grant, and delays in disbursements to subnational levels [5].

A comprehensive study on the economic and fiscal costs of Zambia’s routine immunisation services was undertaken in 2012–13, before introduction of the three new vaccines [6]. This was part of the multi-country ‘Expanded Programme on Immunisation Costing (EPIC)” studies, which used a common, ingredients-based costing approach [7]. The study found that average costs per vaccine dose delivered totalled US$ 7.18, with markedly higher unit costs in rural than urban facilities.

Our study objectives were to estimate the incremental costs of introducing PCV, MSD and RV and evaluate affordability after cessation of Gavi support. Although ‘one-time’ vaccine introduction costs were calculated, the primary objective was to examine the longer-term economic costs.

### 1.1 Gavi eligibility criteria and co-financing policy

Gavi’s current eligibility criteria, established in 2011, is Gross National Income (GNI) per capita of less than US$ 1500, which is adjusted annually for inflation to remain constant in real terms. In 2015, the threshold was US$ 1580 [8]. If GNI per capita increases above the threshold, the recipient country starts transitioning out of support [1].

Gavi’s co-financing policy requires countries to co-procure a portion of their new vaccines and injection equipment. MSD is exempted from co-financing, but after five years countries must take on the full costs [9]. Countries are divided into groups according to GNI per capita, which serves as a proxy for ability to pay [10] (Table 1). The trajectory towards self-financing is achieved by annual increases in co-financing levels in the highest income groups. The ability of countries to shoulder the increasing financing requirements has been questioned and shown to vary substantially [11], [12].

## 2 Methods

### 2.1 Incremental, economic costs of vaccine introductions

Economic costs were estimated in 2014 values, using an exchange rate of 6.18 Zambian Kwacha for one US$ [13] and adjusting earlier data by the Zambian Gross Domestic Product (GDP) deflator [14]. Economic costs were divided into ‘one-time’ and recurring. ‘One-time’ costs were expenditures specifically undertaken in preparation for the new vaccine introductions. Recurring costs were those that occur annually in the future. Capital costs were annualised using a 3% discount rate [15], but also presented without annualisation to show needed up-front expenditures.

The number of fully immunised children was approximated by the number reported to receive three doses of pentavalent vaccine (penta3). With 602,000 surviving infants and 86% penta3 coverage in 2014, this was 517,720 children [16]. Costs per capita were estimated using a 2014 population of 15,023,315 [17].

#### 2.1.1 Vaccine and injection supplies

Vaccine costs were calculated by multiplying price per dose, coverage rate of the first dose, target population size, number of doses per child in the schedule and the vaccine wastage factor [18]. UNICEF 2014 vaccine dose prices were US$ 2.10 for RV, US$ 0.252 for MSD and US$ 7.00/3.50 for PCV [19]. According to the Advance Market Commitment for PCV, a certain quantity of doses is purchased for US$ 7.00 per dose and the remaining at the “tail price” of US$ 3.50 [20]. Since co-financing calculations are based on the tail price and as this will be the price Zambia will pay after Gavi transition, we used this price [21]. Freight charges for importing vaccines were 3%, 5% and 14% of the procurement value for PCV, RV and MSD, respectively [9]. 2014 vaccine coverage rates of the first doses of PCV and RV were assumed similar to DTP1 at 96% while coverage of MSD was 33% [16]. Vaccine wastage rates were assumed as 5% for both PCV and RV and 40% for MSD [22].

#### 2.1.2 Cold storage equipment

Cold storage expansions were undertaken at national, provincial, district and health facility levels [23]. Several development partners contributed to this investment [2]. A proportion of the investments was allocated to the three new vaccines based on their relative packed volumes in the new schedule; 4.8 cm^3^ per dose for PCV, 17.1 cm^3^ for RV and 2.13 cm^3^ for measles [19], [24]. The WHO vaccine volume calculator showed that the new vaccines increased the required volume by 70%, from 81.3 cm^3^ to 138 cm^3^ per penta3 child [25]. The new vaccines occupied 41% of total vaccine volume; PCV took up 11%, RV 25% and MSD 5%.

Power consumption for equipment was collected from WHO Product Information Sheets [26]. A unit price of US$ 0.089 per kilowatt per hour was obtained from ZESCO, the parastatal electricity company. Annual maintenance costs of cold storage equipment were estimated as 10% of the purchase price, based on a UNICEF maintenance grant.

#### 2.1.3 Transport

The EPIC study found that annualised costs of vehicles, vehicle maintenance and fuel comprised 12.5% of total immunisation programme costs, amounting to US$ 4.35 million in 2014 values, or US$ 8.39 per penta3 child [6]. Vaccine delivery comprised a weighted average of 31% of total transport costs (35% in rural and 13% in urban facilities). Hence, vaccine transport costs amounted to US$ 2.60 per penta3 child. We increased the vaccine transport costs by 70%, in line with the increased vaccine volumes (43% due to RV; 18% to PCV; 9% to MSD), amounting to an incremental cost per penta3 child of US$ 1.82.

#### 2.1.4 Training, social mobilisation and communications

As PCV and MSD were introduced concurrently, training and social mobilisation were done at the same time. Based on advice from key informants, we allocated 80% of these costs to PCV and 20% to MSD. Costs were annualised over two years, reflecting the usual frequency of training.

New vaccination cards and health facility registers were printed, the Health Management Information System was modified, and a post introduction evaluation was conducted in July 2014 [27]. These costs were equally distributed between the three vaccines. For PCV, temperature monitoring log sheets and stickers instructing health workers to dispose of unused, opened vials were applied to health facility refrigerators. These investments were all annualised using a useful life of ten years.

#### 2.1.5 Human resources

Introduction of new vaccines did not lead to additional employments, but staff opportunity costs need to be accounted for. In the EPIC study, clinical staff at 51 randomly selected health facilities in nine districts were asked to allocate their working hours among all activities during one month [6]. Costs per dose delivered were estimated by dividing staff immunisation costs by number of vaccine doses delivered per year. Weighted average staff cost per dose was US$ 1.77, ranging from US$ 0.96 in urban to US$ 4.59 in rural facilities [6].

The EPIC study took place before introduction of the new vaccines, but facility staff were asked if they thought introductions would impact operating costs, and 73% responded that they did not think so [6]. However, in the Post Introduction Survey, 17 out of 26 health workers said that the new vaccines had increased their workload [27]. In the absence of time-motion studies, we allocated half of the EPIC study staff cost per dose to RV and PCV (US$ 0.885), as these are delivered at the same time as pentavalent vaccine. However, as MSD requires an additional visit at 18 months, we allocated the full economic cost of US$ 1.77 to each MSD dose delivered [6].

### 2.2 Affordability of Gavi supported vaccines

Zambia’s co-financing contributions were provided by the Gavi secretariat, and the Gavi website gave values of its vaccine and injection supplies donations [28]. Predictions of GNI per capita were used to determine when Zambia enters the accelerated transition phase [29]. Future co-financing amounts were calculated by assuming constant prices per dose of all four Gavi supported vaccines and using data from the United Nations Population Division to predict numbers of surviving infants [30]. Vaccination coverage was assumed constant for PCV and RV, and assumed to increase by 5% per year for MSD. Future costs of the remaining vaccines in the schedule were estimated similarly.

Total government immunisation programme costs were the sum of systems costs, procurement of traditional vaccines and injection supplies, and government expenditure to co-procure vaccines for Gavi co-financing. The EPIC study found that immunisation systems costs amounted to US$ 31.8 million in 2011, equivalent to US$ 61 per penta3 child [6]. Government immunisation costs for 2014 and beyond were determined by adding the incremental systems costs of the new vaccine introductions to this estimate and increasing costs in proportion with the rising birth cohort and, for MSD, also increased coverage.

When assessing affordability, we compared immunisation programme costs with total government health expenditures. For 2008–2014, government health expenditures were collected from the Accountant General’s Office [31]. For 2015, the health sector budget was used, and for 2016 and 2017, the mid-term expenditure framework Green Paper [32], [33]. As the Zambian government considers staff costs fixed, we used government health expenditures excluding personnel costs when evaluating the affordability of vaccines only.

For 2018–2022, we assumed a nominal growth in health care expenditures of 10% per year, which was the average increase during 2014–2016, and equivalent to 5% real, annual increase with predicted inflation rates of around 5% [29]. We also considered a scenario assuming 0% real increase in annual health expenditures.

## 3 Results

### 3.1 Incremental costs of new vaccine introductions

#### 3.1.1 Financial costs

‘One-time’, non-annualised, incremental costs amounted to US$ 4.21 million, equivalent to costs per infant in the birth cohort of US$ 0.96 for MSD, US$ 2.02 for PCV and US$ 3.58 for RV (Table 2). Cold storage rehabilitation and expansion totalled US$ 4.73 million, of which US$ 3.04 million was spent on health facility refrigerators and US$ 1.20 million on national and provincial walk-in cold rooms. Based on the volume estimates, US$ 1.94 million of the total was allocated to the three new vaccines (Table 2). Cold storage investments accounted for 46% of total ‘one-time’ costs while social mobilisation and training accounted for 16% and 14%, respectively.

Cold storage expansions were financed by JICA (36%), ELMA Foundation (34%), Zambian government (10%), Canadian International Development Agency (9%), ARK (7%), WHO (4%), and Boston University (0.3%). Gavi, through the vaccine introduction grant, funded 64% of the social mobilisation, training and monitoring activities. The remainder was paid by the government (29%), GSK (3%), WHO (3%) and Absolute Returns for Kids (1%).

#### 3.1.2 Annualised incremental costs

Annual costs of vaccines and injection equipment increased from US$ 6.8 million to US$ 7.0 million after introduction of MSD, to US$ 14.0 million after PCV, and to US$ 16.9 million after RV (Table 3). Compared to the old schedule, the corresponding increases in vaccine costs were 2% after MSD, 103% after PCV and 147% after all three vaccines were introduced. Vaccine costs per penta3 child were US$ 13 under the old schedule and US$ 33 after the three new vaccine introductions.

When ‘one-time’ costs were annualised, training and social mobilisation were the most important capital costs, each comprising 2% of total costs (Table 4). Cold storage investment only comprised 1% of total vaccine introduction costs after annualisation. The 70% increase in vaccine volume caused extra vaccine transport costs of US$ 941,715 per year. Incremental staff time opportunity costs were US$ 351,628 for MSD, US$ 1.38 million for PCV, and US$ 900,381 for RV.

The EPIC study concluded that economic costs of routine immunisation in Zambia totalled US$ 38.2 million in 2011, equivalent to US$ 66 per penta3 child [6]. The three new vaccines increased total economic costs by an estimated 38% to US$ 52.9 million in 2014, corresponding to US$ 102 per penta3 child. Incremental, annual systems costs of introducing the three new vaccines were US$ 4.9 million, equivalent to US$ 7.69 per child in the birth cohort and US$ 0.31 per capita. The systems costs per vaccine dose administered were US$ 1.33 for PCV, US$ 2.15 for RV and US$ 3.43 for MSD.

### 3.2 Affordability

#### 3.2.1 Co-financing of new vaccines

Zambia introduced pentavalent vaccine in 2005. Gavi’s first co-financing policy required countries to start co-procuring six years after introducing the first new vaccine; thus 2011 for Zambia. With GNI per capita of US$ 490 in 2005, Zambia belonged to the poorest co-financing country group, entailing flat levels of US$ 0.20 per dose for the first vaccine [10]. However, Zambia started voluntary co-financing in 2008 and paid US$ 0.82, US$ 0.92, US$ 0.30 and US$ 0.35 per dose of pentavalent vaccine during 2008–2011, respectively. This meant that the government procured 17% of pentavalent vaccines during those four years.

When Gavi’s second co-financing policy came into effect in 2012, Zambia’s GNI had increased to US$ 1,650 per capita, placing it in the intermediate group, requiring a 15% annual increase in the per dose amount. During 2012, Zambia procured the equivalent of US$ 0.27 per dose in co-financing for pentavalent vaccine and US$ 0.20 for PCV. During 2013, co-financing of RV was added, starting at US$ 0.20 per dose. During 2015, approximately 12% of pentavalent vaccines, 8% of PCV and 9% of RV were co-financed.

Gavi’s new co-financing policy starts in 2016, but the first year is a grace year with the old policy still applying. Zambia is in the accelerated transition group, as its GNI per capita has exceeded the Gavi eligibility threshold for three consecutive years (US$ 1650 in 2012, US$ 1700 in 2013 and US$ 1680 in 2014). Hence, Zambia is predicted to fully finance all vaccines in 2022.

**Fig. 1:**
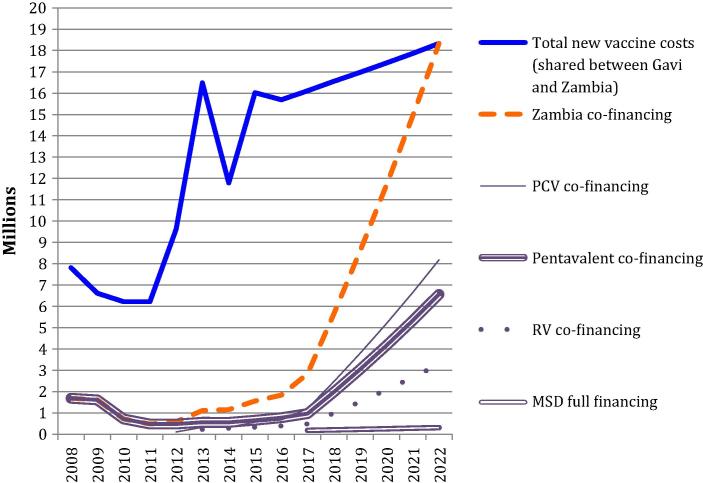
Zambia’s co-financing of new vaccines supported by Gavi, 2008–2022 (US$).

Fig. 1 shows historical and predicted co-financing for the four new vaccines. Co-financing was US$ 1.7 million in 2015 and will increase to the full costs of the vaccines of US$ 18.3 million in 2022. While co-financing on average increased by 10% per year during 2008–2017, it needs to increase by an annual average of 49% between 2017 and 2022. During 2008–2021, vaccines totalling US$ 54.5 million will need to be co-procured, with pentavalent vaccine comprising 42%, PCV 39%, RV 16% and MSD 2%. Total vaccine costs according to financing source between 2008 and 2022 are included in Table A1 in supplementary material.

#### 3.2.2 Affordability assessment

**Fig. 2:**
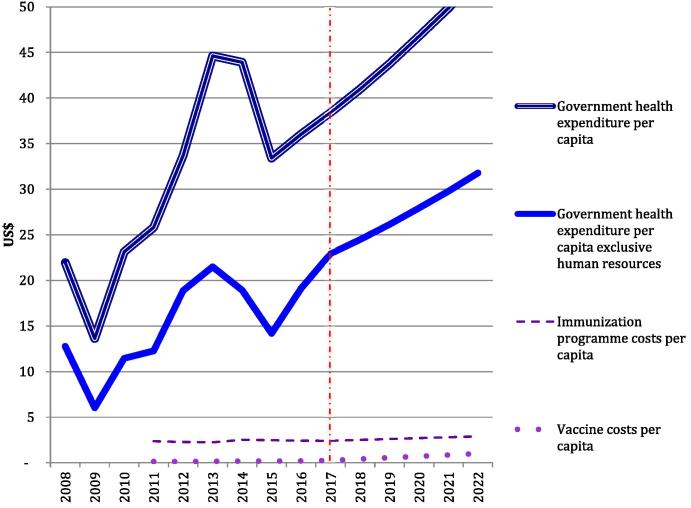
Actual and predicted Government health and immunisation expenditures per capita, 2008–2022.

During 2008–2016, per capita government health expenditures fluctuated between US$ 14 in 2009 and US$ 45 in 2013 (Fig. 2). Between 2009 and 2014, the government increased its real health expenditures (in Kwacha) by an average of 27% per year, but in 2014 and 2015 there was a real decrease of 1% and 2%, respectively. Dramatic depreciation of the Kwacha against the US$ in 2014 and 2015 made this equivalent to decreases of 9% and 23% in US$ terms, respectively. Personnel comprised between 42% and 58% of the government health budgets between 2011–2017. In 2014, government health expenditures inclusive and exclusive of personnel totalled US$ 690 million and US$ 297 million, respectively.

**Fig. 3:**
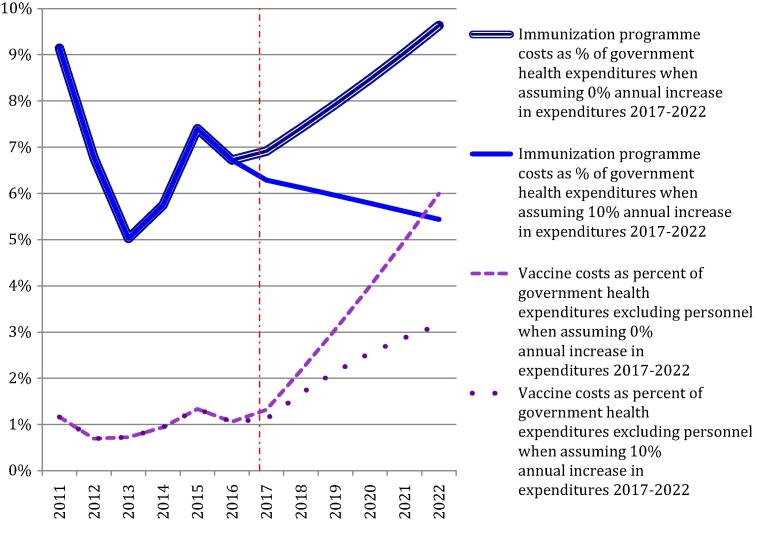
Effect of new vaccine introduction on immunisation programme costs in Zambia.

Routine immunisation programme costs were US$ 52.9 million in 2014, equivalent to 0.2% of GDP. Gavi funded approximately 20% of these costs. Immunisation programme expenditures paid for by the government comprised approximately 6% of government health expenditures. Vaccine expenditures comprised 1% of government health expenditures excluding personnel (Fig. 3). Per capita immunisation programme costs were US$ 2.36 in 2011, US$ 2.54 in 2014 and projected as US$ 2.91 in 2022. Government funded vaccine and injection equipment costs per capita will increase from US$ 0.14 in 2011 to US$ 1.00 in 2022.

Assuming a 5% annual, inflation-adjusted increase in government health expenditures during 2017–2022, immunisation programme costs are projected to be 5% of total health expenditures in 2022. When assuming 0% increase in government health expenditures, immunisation costs would account for 10% in 2022.

Considering vaccine costs alone and assuming 5% real increase in government health expenditures, these will account for 3% in 2022 exclusive personnel. When assuming 0% annual increase until 2022, the proportion is 6%. For vaccine costs to remain at 1% of government health expenditures, excluding human resources, the nominal budget needs to increase by 30% each year between 2017 and 2022.

## 4 Discussion

Incremental, annual systems costs of introducing the three new vaccines were US$ 4.9 million, equivalent to US$ 7.69 per child in the birth cohort and US$ 0.31 per capita. With a total of 2.8 million doses delivered, the systems costs per vaccine dose delivered of all three vaccines were US$ 1.78. The systems costs were US$ 1.33 for PCV, US$ 2.15 for RV and US$ 3.43 for MSD. Costs of simultaneous introduction of PCV, RV and MSD in Ghana in 2012 totalled US$ 2.42 per dose [34]. As this analysis was part of the EPIC studies, the methodology was quite similar to the present Zambia study and differences are likely due to distinct levels of unit costs between the two countries. A study in Gambia found incremental systems costs of PCV introduction of US$ 1.90 [35]. The higher costs in Gambia could be because this was not part of a simultaneous introduction. A study in Rwanda found economic systems costs of introducing PCV and RV of US$ 0.68 and US$ 0.54 per dose, respectively [36]. The higher costs in Zambia than Rwanda are especially due to differences in methods for estimating staff costs. While our estimate was based on economic costing of a sample of health facilities by the EPIC study, the Rwanda study assumed that it took six minutes to deliver a dose of vaccine, based on program manager information.

When including costs of vaccines, annual incremental costs of the three introductions amounted to US$ 23 per child in the birth cohort. It led to a cost increase of 38%, resulting in total immunisation programme costs of US$ 52.9 million in 2014, equivalent to US$ 102 per penta3 child. This is comparable to US$ 132 per penta3 child in Honduras, where PCV, RV and MSD have also been introduced [37].

Cold chain investments may be essential for new vaccines, and have been a major focus of cost studies of earlier new vaccine introductions [3]. However, in Zambia the new equipment only represented 1% of incremental costs over time. Hence, while the ‘one-time’ costs are a substantial up-front investment, they are less important when spread over their expected lifetime.

Government health expenditures have fluctuated since 2008. Zambia experienced a serious economic situation in 2015. The annual economic growth rate declined from an average of 7% since 2010 to 3.4% in 2015 [38], partly caused by a drastic drop in the international copper price, Zambia’s main export earner [39]. It is consequently challenging to predict whether or by how much real government health expenditures will increase in coming years. Moreover, wide fluctuations of the Kwacha increase uncertainty. The average exchange rate to the US$ was 4.79 Kwacha in 2011. In 2012, 2013 and 2014, the rates were 5.18, 5.35 and 6.12, respectively [13]. However, in 2015, the average rate was 8.56 and it reached 10.90 by the end of December, making the Kwacha that year’s worst performing currency against the US$ [13], [38].

Immunisation is projected to comprise 5–10% of government health expenditures in 2022, compared to 6% in 2014. The proportion depends on the extent real government health expenditures increases and assuming no further deterioration in the exchange rate. Even though there are no benchmarks for optimal spending on immunisation, these proportions must be considered relatively high. Projected vaccine and injection equipment costs of 3–6% of non-personnel health spending are also higher proportions than those reported as potentially challenging in other countries [12], [40].

Numerous studies from sub-Saharan Africa have shown substantial health impacts and cost-effectiveness of PCV, RV and MSD [41], [42], [43]. There is thus little doubt that these vaccines are a sound investment in population health, but substantial increases in domestic financing for vaccines are necessary to ensure sustainability. Zambia funded 83% of immunisation programme costs in 2011 and has continued to increase its contribution, showing strong commitment [6]. Increasing resources to health or improving efficiency of services can potentially create fiscal space. However, it is a substantial challenge to achieve reallocations of the magnitude required for the multiple new vaccines and increased provisions to health can be expected to be difficult given the economic context. If increasing provisions to vaccines inadvertently crowd out funding of other items, such as transport for supervision and outreach, this could damage overall immunisation performance. Hence, all sustainability options require advocacy and responsive planning to reinforce the focus of government and partners on ensuring optimal transition.

## Figures and Tables

**Table 1:** Gavi co-financing policies 2008–2017.

	2008–2011	2012–2016	2017–
Country groups	1. Fragile 2. Poorest 3. Intermediate 4. Least poor	1. Low-income 2. Intermediate 3. Graduating	1. Initial self-financing 2. Preparatory transition 3. Accelerated transition
Initial co-financing levels per vaccine dose	▪ US$ 0.10 – US$ 0.30 ▪ Amounts differed for first and subsequent approved vaccines	US$ 0.20	US$ 0.20
Annual increase in co-financing per dose	15% for the least poor group	15% for the intermediate group	15% for the preparatory transition group
Trajectory for transitioning out of support	None specified	Graduating group: ▪ Linear increase to reach full vaccine price after 5 years	Accelerated transition group: ▪ Linear increase to reach full vaccine price after 5 years
Co-financing linked to vaccine price	No link	Graduating group: ▪ Paying linearly towards full vaccine price	Preparatory transition group: ▪ Co-financing for individual vaccine differ according to vaccine prices Accelerated transition group: ▪ Paying linearly towards full vaccine price

**Table 2:** Financial costs of new vaccine introduction in Zambia (2014 US$).

	MSD	PCV	RV	Total	Percent of total
Cold storage investments	228,515	505,663	1,207,044	1,941,223	46%
Social mobilisation	60,962	243,849	377,190	682,001	16%
Training	40,398	161,591	386,824	588,813	14%
Tally sheets and under-5 cards	157,773	157,773	157,773	473,320	11%
Post introduction evaluation	94,778	94,778	94,778	284,335	7%
Monitoring tools	32,876	131,505	76,918	241,300	6%
**Total**	**615,304**	**1,295,161**	**2,300,528**	**4,210,992**	**100%**

Costs per infant in birth cohort	0.96	2.02	3.58	6.56	
Costs per capita	0.04	0.09	0.15	0.28	

MSD: Measles second dose, PCV: Pneumococcal conjugate vaccine, RV: Rotavirus vaccine.

**Table 3:** Vaccine and injection supplies costs with and without measles second dose, pneumococcal, and rotavirus vaccines (2014 US$).

Antigen	Doses per person	Vaccine coverage of first dose	Vial size	Wastage in percent	Costs per dose (incl. freight)	Total vaccine costs	Injection supply costs	Total costs	% of total
Bacille Calmette Guerin	1	95%	20	50%	0.15	185,495	52,669	238,164	1%
Diphteria-Tetanus-Pertussis-HepB-Hib	3	96%	1	5%	2.87	5,242,890	120,314	5,363,205	32%
Measles	1	85%	10	40%	0.29	245,002	40,073	285,075	2%
Oral polio vaccine	4	96%	10	25%	0.23	701,364	–	701,364	4%
Tetanus toxoid	2	74%	10	25%	0.12	172,084	77,881	249,966	1%
**Total before new vaccines**						**6,546,835**	**290,937**	**6,837,773**	

Measles 2nd dose	1	33%	10	40%	0.29	97,564	15,558	115,567	1%
Pneumococcal conjugate	3	96%	2	5%	3.61	6,766,366	120,314	7,073,884	42%
Rotavirus	2	96%	1	5%	2.21	2,757,647	–	2,832,528	17%
**Total with new vaccines**						**16,168,412**	**411,252**	**16,859,752**	**100%**

**Table 4:** Annualised costs of new vaccine introduction in Zambia (2014 US$).

	Measles 2nd dose	Pneumococcal	Rotavirus	Total
*Annualised one-time costs:*				
Training	21,112	84,449	202,158	307,720
Social mobilisation	21,552	107,760	133,348	262,660
Cold storage investments	25,900	55,592	132,702	214,194
Tally sheets and under-5 cards	55,778	55,778	55,778	167,333
Monitoring tools	23,603	58,114	27,193	108,909
Post introduction evaluation	33,507	33,507	33,507	100,521
***Sub total***	***181,451***	***395,200***	***584,686***	***1,161,337***

*Recurring costs:*				
Vaccines	97,564	6,766,366	2,757,647	9,621,577
Human resources	351,628	1,382,538	900,381	2,634,548
Vaccine transport	124,758	241,200	575,757	941,715
Cold storage	23,248	52,172	124,538	199,958
Injection supplies	15,558	120,314	–	135,872
***Sub total***	***612,755***	***8,562,591***	***4,358,323***	***13,533,670***
**Total annualised costs**	**794,206**	**8,957,792**	**4,943,009**	**14,695,007**

2014 reported last dose coverage	33%	77%	73%	NA
Children reached with last dose	198,660	463,540	439,460	NA
Cost per child reached with last dose	4.00	19.32	11.25	NA
Costs per dose delivered	4.00	5.17	4.28	NA
Costs per infant in birth cohort	1.24	13.95	7.70	22.89
Costs per capita	0.05	0.60	0.33	0.98

## Appendix A Supplementary material

Supplementary Table A1
