# Supplementary material for: Costs of introducing pneumococcal, rotavirus and a second dose of measles vaccine into the Zambian immunisation programme: Are expansions sustainable?
Source: Vaccine. 2016 Jul 29;34(35):4213–20. doi: 10.1016/j.vaccine.2016.06.050 (PMC4967451; doi:10.1016/j.vaccine.2016.06.050)
Supplement: Supplementary Table A1 [file mmc1.docx]

**Annex: Table A1: Total vaccine costs of the 2014 Zambian routine vaccination schedule*, 2008-2022 (1000 US$)**

|  | **2008** | **2009** | **2010** | **2011** | **2012** | **2013** | **2014** | **2015** | **2016** | **2017** | **2018** | **2019** | **2020** | **2021** | **2022** |
| --- | --- | --- | --- | --- | --- | --- | --- | --- | --- | --- | --- | --- | --- | --- | --- |
| New vaccines financed by Gavi | 6,130 | 5,003 | 5,501 | 5,737 | 9,037 | 15,374 | 10,634 | 14,465 | 13,856 | 13,275 | 10,900 | 8,373 | 5,721 | 2,930 | - |
| Traditional vaccines financed by Government | 1,251 | 1,286 | 1,322 | 1,358 | 1,396 | 1,435 | 1,475 | 1,514 | 1,554 | 1,594 | 1,636 | 1,675 | 1,717 | 1,759 | 1,803 |
| Gavi co-financing funded by Government | 1,684 | 1,612 | 581 | 685 | 542 | 941 | 1,318 | 1,556 | 1,837 | 2,836 | 5,656 | 8,603 | 11,699 | 14,936 | 18,335 |
| **Total vaccine costs** | **9,065** | **7,901** | **7,403** | **7,780** | **10,975** | **17,750** | **13,426** | **17,535** | **17,247** | **17,705** | **18,192** | **18,651** | **19,138** | **19,625** | **20,139** |
| Percent financed by the Government | 32% | 37% | 26% | 26% | 18% | 13% | 21% | 18% | 20% | 25% | 40% | 55% | 70% | 85% | 100% |

* Zambia introduced inactivated polio vaccine in 2015. This and any other future vaccines, such as for instance human papilloma virus vaccine, are not included in the calculations. Vaccines procured for campaigns are not included either.
